# Comparison of airway measurements during influenza-induced tachypnea in infant and adult cotton rats

**DOI:** 10.1186/1471-2466-9-28

**Published:** 2009-06-10

**Authors:** Elman L Trias, Arash Hassantoufighi, Gregory A Prince, Maryna C Eichelberger

**Affiliations:** 1 Division of Allergy, Pulmonary & Sleep Medicine, Children's National Medical Center, Washington, DC 20010, USA; 2 Division of Viral Products, Center for Biologics Evaluation and Research, Food and Drug Administration, Bethesda, MD 20892, USA; 3 Virion Systems Inc, Rockville, MD 20850, USA; 4 Current address: Kaiser Permanente, 2025 Morse Avenue, Sacramento, CA 95825, USA

## Abstract

**Background:**

Increased respiratory rate (tachypnea) is frequently observed as a clinical sign of influenza pneumonia in pediatric patients admitted to the hospital. We previously demonstrated that influenza infection of adult cotton rats (*Sigmodon hispidus*) also results in tachypnea and wanted to establish whether this clinical sign was observed in infected infant cotton rats. We hypothesized that age-dependent differences in lung mechanics result in differences in ventilatory characteristics following influenza infection.

**Methods:**

Lung tidal volume, dynamic elastance, resistance, and pleural pressure were measured in a resistance and compliance system on mechanically-ventilated anesthestized young (14–28 day old) and adult (6–12 week old) cotton rats. Animals at the same age were infected with influenza virus, and breathing rates and other respiratory measurements were recorded using a whole body flow plethysmograph.

**Results:**

Adult cotton rats had significantly greater tidal volume (TV), and lower resistance and elastance than young animals. To evaluate the impact of this increased lung capacity and stiffening on respiratory disease, young and adult animals were infected intra-nasally with influenza A/Wuhan/359/95. Both age groups had increased respiratory rate and enhanced pause (*Penh*) during infection, suggesting lower airway obstruction. However, in spite of significant tachypnea, the infant (unlike the adult) cotton rats maintained the same tidal volume, resulting in an increased minute volume. In addition, the parameters that contribute to *Penh *were different: while relaxation time between breaths and time of expiration was decreased in both age groups, a disproportionate increase in peak inspiratory and expiratory flow contributed to the increase in *Penh *in infant animals.

**Conclusion:**

While respiratory rate is increased in both adult and infant influenza-infected cotton rats, the volume of air exchanged per minute (minute volume) is increased in the infant animals only. This is likely to be a consequence of greater lung elastance in the very young animals. This model replicates many respiratory features of humans and consequently may be a useful tool to investigate new strategies to treat respiratory disease in influenza-infected infants.

## Background

Influenza continues to cause major epidemics worldwide. The highest infection and hospitalization rates are observed in infants and young children [1]. Among this group, hospitalization rates are inversely related to the age of the child, being highest in those younger than 6 months. The absence of prior immunity and the immaturity of the infant's immune system upon exposure to the virus are potential contributors to their increased risk of severe disease [2]. Epidemiologic studies indicate that children with certain chronic conditions including asthma, and otherwise healthy children younger than 24 months are at high-risk of being hospitalized after influenza infection. Complications of influenza in these groups are similar to those experienced by the elderly [3]. There is however, still no influenza vaccine licensed for use in infants younger than 6 months and there are no antivirals licensed for the treatment of influenza in infants younger than 1 year of age.

The clinical presentation of influenza varies in children [1]. In general, after an incubation period of 1–4 days, it is characterized by fever, myalgia, headache, non-productive cough, rhinitis and sore throat. Other manifestations in infancy include irritability, decreased oral intake, vomiting, diarrhea, abdominal pain and lethargy. More severe illness can result if there is lower airway involvement usually secondary to pneumonia, resulting in difficulty breathing, apnea and more frequently, tachypnea. Mechanical ventilatory support is necessary in severe illness. This supportive care is likely to be essential for recovery of very young infants in whom physiologic mechanisms that allow passive expiration have not yet developed. In older children and adults, passive expiration occurs due to recoil of the inflated lung against the chest wall. In the very young, the lung has the ability to distend to a greater degree because the chest wall has not stiffened [4] and lung recoil is therefore not possible. Animal models that accurately represent age-dependent differences in lung mechanics may be a useful tool to investigate new treatment strategies to treat lower respiratory tract infections in infants.

The cotton rat is a suitable model to study several human respiratory viruses. This species is permissive for the replication of respiratory syncytial virus [5], parainfluenza virus [6], adenovirus [7], measles virus [8], and influenza virus [9]. We have used adult cotton rats to examine the effect of influenza virus infection on lung pathology, demonstrating that virus replicates in the lung and nose of an infected cotton rat for 2 and 7 days respectively, resulting in considerable epithelial cell destruction and inflammation in the lower respiratory tract, as well as increased respiratory rates [9,10].

We used a whole-body flow plethysmograph (WBP) for small animals that allows non-invasive, repeated assessments of pulmonary physiological parameters to evaluate ventilation in influenza virus-infected adult cotton rats. Infection increased the animal's respiratory rate. The host mechanism that controls the tachypneic response has not been defined; tachypnea does correlate with inoculum dose and requires the presence of live virus [10]. This suggests that the host responds to the first round of replication, perhaps through sensing excessive tissue damage that is a result of oxygen or nitrogen free radicals, innate mediators or cell death. Oxidized phosholipid present during lung infection or injury may trigger a response through TLR4 [11]. Alternatively, it has been demonstrated that the lung rennin-angiotension system plays an essential role in protecting the lung and is known to play a role during pandemic influenza infection [12]. Sensory neurons are also likely to contribute to induction of tachypnea in order to maintain an appropriate oxygen/carbon dioxide balance.

We hypothesized that age-dependent differences in lung mechanics would result in different ventilatory characteristics following influenza infection of adult and infant animals. This was indeed the case: our results show that lung elastance and relative lung capacity are greater in 14 day old animals than adults. These observations explain the differences in respiratory measurements recorded for influenza-infected infant and adult cotton rats.

## Methods

### Cotton rats

Male and female inbred *Sigmodon hispidus *were obtained from a breeding colony maintained at Virion Systems Inc. (Rockville, MD) or were purchased from ACE Animals (Boyertown, PA). All animal experiments were performed following federal guidelines using protocols approved by the Institutional Animal Care and Use Committee. Animals were seronegative for adventitious viruses. Infant 14-day old cotton rats (20 ± 2 g) were used and compared with adult 6 to 12 week old cotton rats (weight 80–140 g). We considered 14-day old cotton rats to be infants since they do not spontaneously wean at this age [13]. In our experiments these infants were infected at exactly 14 days age and housed without the mothers. Crushed food was made into a slurry and placed within easy reach of these young animals to ensure their well-being.

### Virus infection and titration

A large pool of A/Wuhan/359/95 was grown in MDCK cells at Dyncorp (Rockville, MD) resulting in a 50% tissue culture infectious dose (TCID_50_) of 10^8^/ml stock virus. Virus was stored at -70°C, thawed immediately before use and diluted in phosphate-buffered saline. Animals were anesthetized in 3% isoflurane and then inoculated intranasally (i.n.) with stock virus using 200 μl/100 g animal. Control animals were inoculated with 200 μl/100 g of a "mock" preparation. This consisted of the supernatant of MDCK cells that were treated in an identical manner (except for virus inoculation) to those that were used to prepare the virus stock. Lungs were surgically removed from animals that were sacrificed by CO_2 _asphyxiation, and homogenized in DMEM supplemented with 0.218 M sucrose-4.9 mM glutamate-3.8 mM KH_2_PO_4 _(1 ml/100 mg tissue). The cellular debris was pelleted and supernatant stored at -70°C for virus titration. Influenza virus titers were determined on monolayers of MDCK cells as previously reported [14]. The titer assigned was the inverse dilution that resulted in cytopathic effect in 50% of infected cell monolayers and reported as TCID_50_/ml of lung homogenate. The limit of detection in this assay is 10^1.8 ^TCID_50_/ml.

### Respiratory measurements

A whole body flow plethysmograph (Buxco Electronics Inc., Wilmington, NC) was used to directly measure respiratory rate (*f*) in breaths per minute (bpm), tidal volume (TV), minute volume (MV), time of expiration (Te), relaxation time (RT), peak inspiratory flow (PIF) and peak expiratory flow (PEF). After calibration of the 2-chamber equipment, one cotton rat was placed in a small plexiglass container within each plethysmograph chamber and measurements were made over a period of 5 minutes. Prior experiments had established that reproducible results were obtained within this time period. Since recordings were made over short time periods, box humidity and temperature was set at that recorded within the room. The instrument settings for animal body temperature was set at 37.5°C, the average temperature of an adult cotton rat infected with influenza 2 days earlier (normal body temperature measured with a rodent rectal thermometer with thermocouple probe is 39.5°C). Bias flow was set at 2 liters/minute, resulting in a flow past the animals of 1.2 ml/sec. With these conditions the noise level was minimal and the waveform of both the infant and adult animals was distinct. Other parameters of ventilation were derived using the method of Epstein [15], including TV, pause and enhanced pause (*Penh*). The instrument software calculated Pause, a measure of the time it takes to expire the remaining 36% of the total expiratory flow that reflects "effort" of breathing (pause = (Te - RT)/RT) and *Penh*, a dimensionless measure that combines both time and flow rates to describe ventilation (*Penh *= (pause) (PEF/PIF)) [16].

Lung dynamic compliance (C_dyn_), resistance (R), tidal volume (TV) and change in pleural pressure (dPp) were measured using a FinePointe-series all-in-one Resistance/Compliance (RC) system (Buxco Electronics). The system was calibrated for air flow and air pressure. To obtain measurements in the RC system, cotton rats of different ages were anesthetized with ketamine/xylazine. After tracheotomy, the animals were intubated with a sterile cannula that was held in place with surgical thread, and then connected to the ventilator with a fixed breathing rate of 145 bpm. Airway measurements were recorded over a 3 min period using Biosystem XA data acquisition and analysis software. Dynamic elastance was calculated as 1/C_dyn_. Animals were euthanized following an IACUC approved protocol at the completion of the experiment.

### Histopathologic examination of lungs

Lungs were fixed in 10% formalin and hematoxylin and eosin stained sections prepared by Histoserv (Rockville, MD). Stained slides were examined for destruction of epithelium in the small and large airways (epithelial cell damage), the presence of cells or cellular debris in the airways (exudates), infiltration of inflammatory cells around the periphery of small airways (peribronchiolitis), cells within the alveolar walls resulting in alveolar thickening (interstitial pneumonitis), or cells within the alveolar spaces (alveolitis) and around blood vessels (perivasculitis). Each parameter was scored blind on a scale of 0 to 4 (0 being representative of no histopathology and 4 being representative of extensive histopathology) as previously described [9,10], and then transformed to a scale that reflects the degree of pathology as a percent of maximum pathology i.e. a score of 4 that reflects the maximum amount of pathology possible, is shown as 100%.

### Statistical methods

Data are expressed as the mean ± standard error of the mean (SEM). The effects of virus infection on ventilatory measurements were analyzed by paired samples t tests within each age group. The effects of virus infection on histopathology were measured by independent samples t tests at each time point. Proportions of infected animals were compared using the Chi-square test. Statistical analysis was performed using SPSS version 12.0.1 (SPSS Inc., Chicago, IL). Probability values (p) < 0.05 were considered statistically significant.

## Results

### Comparison of ventilatory measurements in young and adult cotton rats

**Figure 1:**
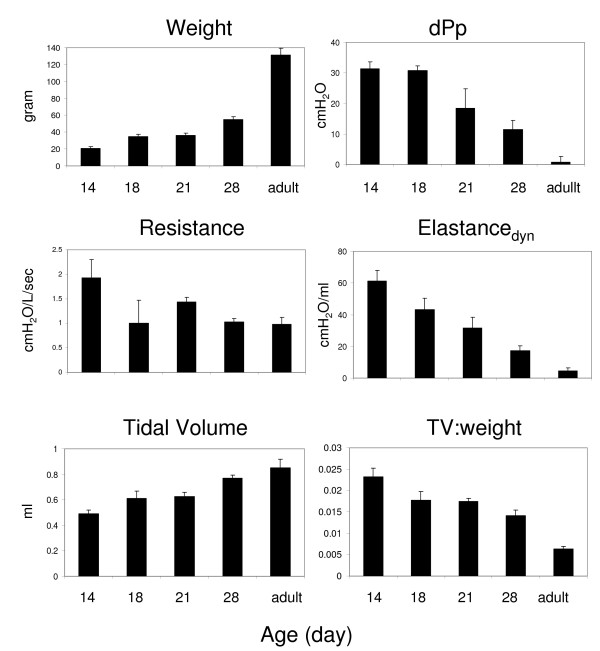
**Comparison of ventilatory parameters in cotton rats at different ages**. The graphs show the average (±SEM) values for 4 animals that were 14, 18, 21, and 28 days old in addition to 11 week old adult animals. Each animal's weight, tidal volume (TV), dynamic elastance (Elastance_dyn_), change in pressure (dPpl), airway resistance, and the ratio of TV and weight was recorded.

Age-dependent changes in ventilatory parameters reflect differences in size of the airways and physiological changes that impact lung elasticity and recoil. We measured tidal volume (TV), dynamic compliance (C_dyn_), dynamic elastance, resistance and change in pleural pressure in adult cotton rats as well as animals that were 14, 18, 21 and 28 days old. As expected, at a set respiratory rate, TV and C_dyn _(dynamic compliance, the change in volume divided by pressure change during inspiration) increased with age; resistance and pleural pressure in the lower airway decreased with age (Figure 1). Interestingly, the increase in tidal volume was not proportional to the increase in weight, resulting in an age-dependent decrease in the TV:weight ratio that mimicked the decrease in dynamic elastance (Figure 1).

### Ventilatory measurements after infection with A/Wuhan/359/95

**Figure 2:**
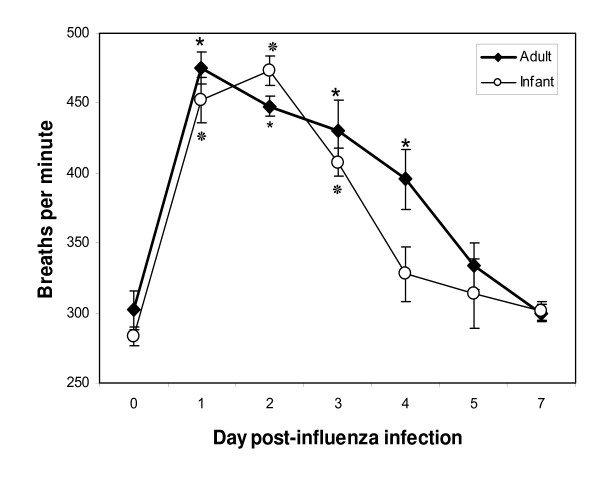
**Respiratory rates (*f*) of cotton rats infected with A/Wuhan/359/95**. The arithmetic means of *f *in infant cotton rats (open circles) are shown and compared to adult cotton rats (closed diamonds) on days 0 (before infection), 1, 2, 3, 4, 5 and 7 p.i. Four to ten animals were used in each group. The SEM is shown for each group. Means that were significantly different from *f *measured on day 0 (t test within in each age group, p < 0.05) are indicated with an * for adults and ^❊^ for infants.

We compared ventilatory parameters that were recorded before infection (day 0 baseline WBP) and on days 1, 2, 3, 4, 5 and 7 post-infection (p.i.) with live influenza virus. The arithmetic mean of each parameter was calculated for each group of cotton rats (at least 4 animals in each group). A significant increase in respiratory rate (*f*) (paired samples t test, p < 0.05) was evident in both infant and adult cotton rats on days 1, 2 and 3 p.i. (Figure 2). As expected from our published results [10], control infant and adult animals that were inoculated with an equivalent volume of 'mock' did not exhibit significant changes in respiratory rate. A small difference 2 days after mock inoculation (*f *before inoculation 287 ± 4 breaths/min; *f *after inoculation 305 ± 13 breaths/min) was not statistically significant (p = 0.06). Both infant and adult influenza-infected cotton rats had greater than 65% increase in *f *compared to baseline on day 1 p.i. Infant cotton rats continued to be more tachypneic on day 2 p.i. whereas the respiratory rate of the adult cotton rats began to decline. Although the infant cotton rats had a longer duration of peak tachypnea (days 1 and 2), resolution was more rapid compared to adults in which *f *continued to be significantly elevated at day 4 p.i. (p < 0.05).

As expected, baseline TV was smaller in infant than adult cotton rats, reflecting the difference in size of the animals. However, in adult animals, TV decreased significantly during tachypnea, while in infant animals, no significant difference was recorded in TV measured before and after influenza infection. Table 1 compares TV and other ventilatory parameters on day 2 post-infection, the day at which respiratory rate peaked in the young animals. This resulted in a significant increase in MV in the infant but not the adult animals after infection. Inspiration and expiration of the same tidal volume in a shorter period of time results in significant increases in both PEF and PIF in infant cotton rats (paired samples t test, p < 0.05). However, the increase in PEF was greater than the increase in PIF resulting in an increased PEF/PIF ratio that contributes to the greater *Penh *that is calculated. In contrast, tachypneic adults had reduced TV and consequently PIF was not significantly increased in this age group. The increase in PEF in adult animals was small compared to the increase in infant cotton rats (approximately 30% and 90% increase after infection in adult and infant cotton rats, respectively).

**Table 1:** Infant and adult ventilatory measurements* before (day 0) and 2 days after infection with A/Wuhan/359/95

	Infants		Adults	
	Day post-infection		Day post-infection	
		
	0	2	0	2
f (bpm)	287 (±24)	473 (±29)^†^	290 (±22)	448 (±21)^†^
TV (ml)	0.169 (±0.036)	0.158 (±0.023)	0.531 (±0.138)	0.381 (±0.056)^†^
MV (ml/min)	48.14 (±10.68)	74.451 (±8.44)^†^	153.542 (±43.47)	169.687 (±22.50)
PIF (ml/s)	2.942 (±0.561)	4.049 (±0.426)^†^	8.799 (±2.574)	9.071 (±1.328)
PEF (ml/s)	2.378 (±0.433)	4.313 (±0.411)^†^	6.733 (±1.821)	8.71 (±0.966)
RT (s)	0.086 (±0.009)	0.041 (±0.003)^†^	0.087 (±0.008)	0.046 (±0.002)^†^
Te (s)	0.121 (±0.011)	0.065 (±0.004)^†^	0.116 (±0.01)	0.068 (±0.005)^†^

*Measurements shown include: Frequency (f) in breaths per minute (bpm), Tidal Volume (TV), Minute Volume (MV), Peak Inspiratory Flow (PIF), Peak Expiratory Flow (PEF), Relaxation Time (RT), Expiratory Time (Te). Infant and adult cotton rats were infected with influenza virus as described in the Materials and Methods. The results shown are the average of 4 – 10 animals, with standard deviation shown in parentheses. Significant differences (paired samples t test, p < 0.05) between day 0 and day 2 are marked with a †.

**Figure 3:**
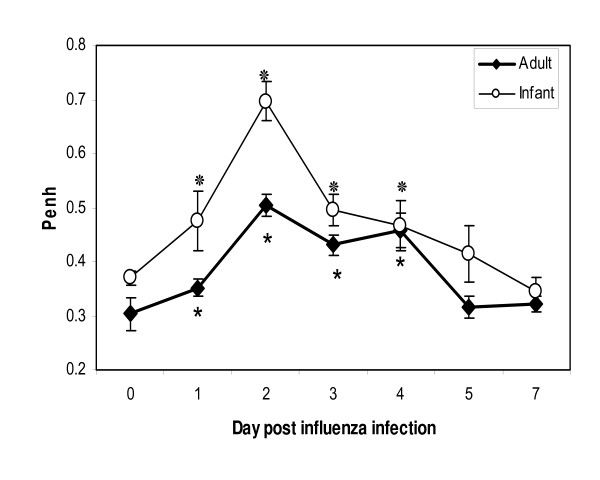
***Penh *of cotton rats infected with A/Wuhan/359/95**. The arithmetic means of *Penh *in infant (open circles) and adult (closed diamonds) cotton rats are shown on days 0 (before infection), 1, 2, 3, 4, 5 and 7 p.i. The SEM is shown for each group and means that were significantly different from *Penh *measured on day 0 (t test within in each age group, p < 0.05) are indicated with an * for adults and^ ❊^ for infants.

In both adult and infant cotton rats, *Penh *was elevated on day 1 p.i. but peaked on day 2, with an increase from baseline of approximately 70% and 85% in adults and infants, respectively (Figure 3). Compared to Penh values measured before infection, elevated *Penh *measurements returned to baseline 5 days later.

### Viral titers in lung homogenates

Influenza infection of the lungs was confirmed in all influenza-inoculated cotton rats (Table 2). Influenza was recovered on day 1 p.i. in both infant and adult animals. Virus cleared after day 2 p.i. in the infant animals, but persisted in one of four adult animals until day 3 p.i. However, since a small number of animals was used, this difference in virus clearance was not statistically significant (chi-square = 3.0).

**Table 2:** Kinetics of A/Wuhan/359/95 replication in cotton rats

Cotton rats	Day p.i.	Geometric mean virus titer ± SEM (TCID_50_/ml)	% animals with detectable virus
Infant	1	10^4.95 ± 0.21^	100
	2	10^2.40 ± 0.29^	75
	3	<10^1.8 ± 0^	0
	4	<10^1.8 ± 0^	0
	7	<10^1.8 ± 0^	0
			
Adult	1	10^5.50 ± 0.20^	100
	2	10^2.40 ± 0.29^	75
	3	10^2.10 ± 0.30^	25
	4	<10^1.8^	0
	7	<10^1.8^	0

### Histopathology of lung tissue

**Figure 4:**
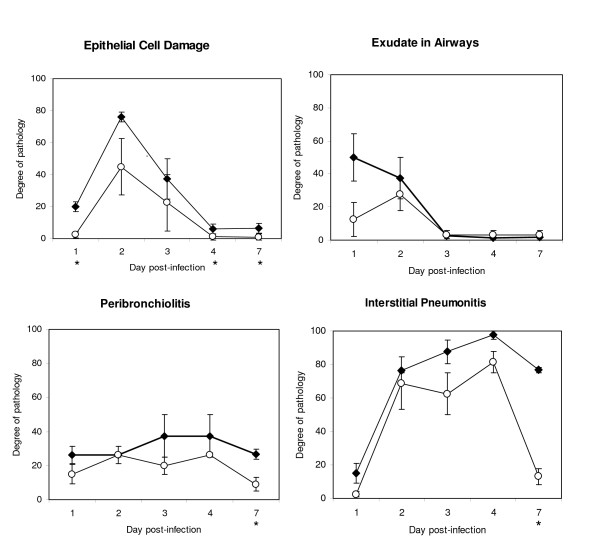
**Pulmonary pathology in the infant (open circles) and adult (closed diamonds) cotton rats infected with A/Wuhan/359/95 on days 1, 2, 3, 4 and 7 p.i**. Results are reported as arithmetic means of histopathology scores that have been transformed to a scale of 0 to 100, reflecting the degree of epithelial cell damage, amount of exudate present in the small and large airways, and the extent of peribronchiolitis and interstitial pneumonitis observed in hematoxylin and eosin-stained lung sections, for 4 animals per group. SEM is indicated at each time point. The time points at which the degree of pathology was significantly different (independent samples t test, p < 0.05) between adult and infant lungs are indicated with an asterisk (*) beneath the x axes.

**Figure 5:**
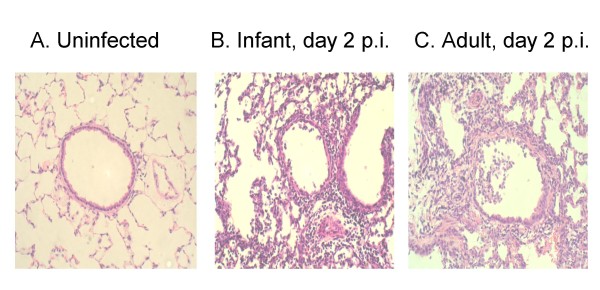
**Representative hematoxylin and eosin-stained sections (× 200) of formalin-fixed lungs from uninfected (A) and 2 days after infection with A/Wuhan/359/95 infant (B) and adult (C) cotton rats**. Significant epithelial cell damage and exudate as well as severe alveolitis and interstitial pneumonitis are observed in the lungs of both infant and adult animals at day 2 post-infection.

Figure 4 shows the degree of pathology observed in the infected cotton rat lungs. There was a trend for epithelial cell damage to be greater in adults than in infants over a 1 week period post inoculation, with the biggest difference noted at early time points. Both age groups had exudates in small and large airways on day 1 and 2 that resolved on day 3 p.i. Interstitial pneumonitis was severe at times of peak tachypnea but persisted and remained significant in adult lungs even at time points after the resolution of tachypnea (Figure 4). Alveolitis scores (not shown) were similar to that of interstitial pneumonitis. Peribronchiolitis (Figure 4) and perivasculitis (not shown) were observed early after infection as well as at time points after the resolution of tachypnea. These inflammatory responses were similar in both the infant and adult animals at early time points but were statistically greater in adult cotton rats on day 7. In figure 5, hematoxylin and eosin-stained lung sections from animals that were uninfected or infected 2 days previously with influenza virus, show each of these pathologic features.

Although the presence of tachypnea did not coincide exactly with any one pathologic feature, the results suggest an association with epithelial cell damage that, like tachypnea, was most severe on days 1, 2 and 3 post-infection. Infection of animals at each age group with an identical dose of virus (5 × 10^6 ^TCID_50_/animal i.e. adults were inoculated with 4-fold less virus than in the experiment shown in the figures) also resulted in peak epithelial cell damage on day 2 p.i. that coincided with peak tachypnea. As expected for a lower inoculum dose, epithelial cell damage was less severe in adults under these latter conditions than in infant animals inoculated with the same dose of virus.

## Discussion

In this study, we present a rodent model to demonstrate age-dependent differences in ventilation after infection with A/Wuhan/359/95. As expected, and reported for animal models as well as humans, our studies show that cotton rat airway tidal volume increases with age, reflecting changes in lung structure, primarily increased diameter of airways and alveoli. For the same reasons, there was a decrease in airway resistance with age. While tidal volume and body weight increased with age, these parameters did not increase at the same rate, resulting in a decrease in TV:weight ratio with age. There was a similar decrease in lung elastance. This may reflect age-dependent physiologic changes such as airway and diaphragm smooth muscle stiffening and developmental changes in the chest wall [4] that result in limited airway dilation in adult animals compared to infants [17-19]. While this may seem a disadvantage, the benefit is immense as the subsequent work of breathing is reduced due to efficient lung recoil against the stiffened chest wall.

To evaluate the impact of age-dependent changes in the airway on respiratory disease, we compared ventilatory parameters in young and adult cotton rats after influenza infection. Tachypnea and lower airway obstruction were evident in both infant and adult cotton rats early after infection and resolved soon after the virus was cleared from the lower respiratory tract, suggesting a direct effect of viral replication on ventilation that correlated with significant epithelial cell damage and the presence of airway exudates. These alterations in ventilation are consistent with clinical changes seen in influenza-infected human infants, in whom tachypnea is a characteristic finding that can be directly observed. These results also validate respiratory rate as an objective measure of respiratory tract disease especially in influenza infection.

Our study confirms the relative ease and non-invasive use of WBP in evaluating ventilation in cotton rats. Influenza clearly induced changes in ventilation as shown by the parameters that were measured. While tachypnea was induced by influenza in both adult and infant animals, there was a decrease in TV in adults but not in infant animals. Consequently, the MV in infected adult animals was unchanged whereas significan MV increases were measured in infected infant animals. Since the total volume of air exchanged per minute is the product of TV and the number of breaths per minute (*f*), this difference in MV between the two age groups suggests that unlike adults, infant cotton rats have the ability to accommodate the same volume of air in their lungs during infection in spite of the influenza-induced obstruction. This difference is likely due to greater lung elastance in very young animals. In addition, the immature central respiratory drive or neuromuscular transmission of infants [20] may contribute to the differences in MV recorded after influenza infection in infant and adult cotton rats. An example of the contribution of sensorineural stimulation that results in an altered breathing pattern is that of respiratory syncytial virus (RSV)-induced apnea in weanling rats [21]. This observation is age-dependent and correlates with number of vagal C fibers [22]. These observations model RSV associated apnea in young infants. Although apnea can be observed after influenza infection [23], it is not a common clinical sign of disease. The equivalent increase in respiratory rate following influenza infection in infant and adult cotton rats suggests that CO_2_/O_2 _sensors and synaptic interactions that control respiratory rate are similar in these age groups. It is therefore logical to propose that structural/mechanical attributes of the infant lung allow a constant tidal volume in influenza-infected infant cotton rats even though there is extensive epithelial cell damage and airway obstruction. Our results show an age-dependent decrease in dynamic lung elastance (ability of lungs to distend); this correlates with the inability of adult influenza-infected cotton rats to maintain equivalent tidal volume in the presence of airway obstruction.

Lung elastance is inversely related to ventilatory efficiency as the alveoli not only expand more easily but also are susceptible to collapse, particularly during forced expiration [19]. The elastance of infant lungs is thought to contribute to sudden infant death syndrome [24]. Therefore, even though it may seem advantageous for infant lungs to accommodate a larger volume when there is airway obstruction, this has risks. We predict that this may also contribute to disease severity in influenza-infected infants. While the stiffness of the chest wall in older children and adults may prevent an increase in TV, this benefits them in that expiration can occur passively as a consequence of lung recoil against the chest wall, limiting the energy required for air exchange and reducing muscle fatigue [25,26]. We therefore hypothesize that tachypneic infants are likely to experience respiratory muscle fatigue, with severe consequences unless they receive mechanical ventilation.

Both infant and adult cotton rats had increased *Penh *values following infection. However, in addition to the contribution of an increase in pause (resulting from decreased RT), the disproportionate increase in PEF (resulting in an increased PEF/PIF ratio) also contributed to the increase in *Penh *in influenza-infected infant, but not adult, cotton rats. In adults, the volume of air retained at the end of expiration is determined by the outward recoil of the chest wall and the lung's inward recoil, whereas young infants use diaphragm and laryngeal adductor activities to control expiratory flow [27,28]. Once the chest wall stiffens and can resist the inward lung recoil force, expiration is maintained passively [4]. Based on these mechanisms, it is likely that the control of expiration in infant cotton rats is different from adult animals and therefore the volume of air, presence of obstruction, as well as decreased compliance, contribute to the increase in PEF observed.

The most obvious and clinically relevant change in ventilation following influenza infection in cotton rats is the tachypnea seen in both age groups. Tachypnea as well as increased *Penh *coincided with epithelial cell damage. Epithelial cell damage is the consequence of virus-induced apoptosis and necrosis and is therefore proportional to the inoculating dose [10]. When infant and adult animals were infected with the same virus dose per 100 g, tachypnea was evident for a longer period of time in adults than infants. This may be the result of the four times greater amount of virus that adults received. On the other hand, when both age groups were infected with an identical dose of virus (5 × 10^6 ^TCID_50_/animal), the adult animals cleared the virus faster than the infant animals. The tachypnea as well as epithelial damage in the adults was less severe and resolved by day 3 p.i. showing that the quantity of the viral load is proportional to the amount of epithelial damage and the increase in respiratory rate.

In contrast to epithelial cell damage, interstitial pneumonitis, peribronchiolitis, perivasculitis and alveolitis (not shown) persisted after tachypnea had resolved. Our findings of influenza-induced tachypnea therefore suggest that epithelial cell damage rather than inflammation plays a crucial role in inducing pulmonary ventilation deterioration. In contrast, tachypnea that is induced in mice following infection with a high dose of RSV coincides with the presence of inflammatory cells. This tachypnea is partially resolved in animals that are deficient in IFN-γ [29], suggesting that the inflammatory response causes the increased respiratory rate following RSV infection.

Age-dependent differences in respiratory parameters support the need for different treatment strategies in influenza-infected infant and adults. Since greater lung elastance measured in infants is inversely related to ventilatory efficiency and could result in alveoli collapse, it may be particularly important to limit influenza infection and reduce the extent and duration of tachypnea in this very young age group.

## Conclusion

Our studies demonstrate age-dependent differences in cotton rat ventilation during influenza infection. Tachypnea is induced in both infant and adults, but the increased lung elastance in infants allows an increase in air exchange per minute (minute volume). Since young cotton rats demonstrate age-dependent measurements similar to those predicted in human infants, studies of respiratory illness by whole body plethysmography in this animal model may contribute important information to our understanding of influenza virus pathogenesis and provide a valuable end-point to evaluate the therapeutic effects of antiviral agents in this age group.

## Abbreviations

bpm: breaths per minute; C_dyn_: dynamic compliance; dPp: change in pleural pressure; f: respiratory rate; MV: minute volume; PEF: peak expiratory flow; Penh: enhanced pause; PIF: peak inspiratory flow; R: resistance; RSV: respiratory syncytial virus; RT: relaxation time; SEM: standard error of the mean; TCID_50_: 50% tissue culture infectious dose; Te: time of expiration; TV: tidal volume; WBP: whole-body flow plethysmograph.

## Competing interests

GAP is President and CEO of Virion Systems, Inc., a biotechnology company that performs contract research using cotton rats. ELT, AH and MCE have no competing interests.

## Authors' contributions

ELT designed the experiments, executed experiments and analyzed data. AH performed airway measurements. GAP assisted in study design and examined lung sections for pathology. MCE designed experiments, performed data analysis and drafted the manuscript. All authors reviewed and approved the final manuscript.

## Pre-publication history

The pre-publication history for this paper can be accessed here:



## References

[B1] Olshaker JS (2003). Influenza. Emerg Med Clin North Am.

[B2] Munoz FM (2003). Influenza virus infection in infancy and early childhood. Paediatr Respir Rev.

[B3] American Academy of Pediatrics, Committee on Infectious Disease (2002). Reduction of the influenza burden in children. Pediatrics.

[B4] Papastamelos C, Panitch HB, England SE, Allen JL (1995). Developmental changes in chest wall compliance in infancy and early childhood. J Appl Physiol.

[B5] Prince GA, Jenson AB, Horwsood RL, Camargo E, Chanock RM (1978). The pathogenesis of respiratory syncytial virus infection in cotton rats. Am J Pathol.

[B6] Murphy TF, Dobovi EJ, Clyde WA (1981). The cotton rat as an experimental model of human parainfluenza virus type 3 disease. Experimental Lung Research.

[B7] Pacini DL, Dubovi EJ, Clyde WA (1984). A new animal model for human respiratory tract disease due to adenovirus. J Infect Dis.

[B8] Wyde RP, Ambrose MW, Voss TG, Meyer HL, Gilbert BE (1992). Measles virus replication in lungs of hispid cotton rats after intranasal inoculation. Proc Soc Exp Biol Med.

[B9] Ottolini MG, Blanco J, Porter D, Peterson L, Curtis S, Prince G (2003). Combination anti-inflammatory and antiviral therapy of influenza in a cotton rat model. Pediatr Pulmonol.

[B10] Eichelberger MC, Prince GA, Ottolini MG (2004). Influenza-induced tachypnea is prevented in immune cotton rats, but cannot be treated with an anti-inflammatory steroid or neuraminidase inhibitor. Virology.

[B11] Imai Y, Kuba K, Neely GG, Yaghubian-Malhami R, Perkmann T, van Loo G, Ermolaeva M, Veldhuizen R, Leung YH, Wang H, Liu H, Sun Y, Pasparakis M, Kopf M, Mech C, Bavari S, Peiris JS, Slutsky AS, Akira S, Hultqvist M, Holmdahl R, Nicholls J, Jiang C, Binder CJ, Penninger JM (2008). Identification of oxidative stress and Toll-like receptor 4 signaling as a key pathway of acute lung injury. Cell.

[B12] Imai Y, Kuba K, Penninger JM (2008). The discovery of angiotensin-converting enzyme 2 and its role in acute lung injury in mice. Exp Physiol.

[B13] Prince G, Zak O, Sande MA (1999). The cotton rat as a model of Respiratory Syncytial Virus pathogenesis, prophylaxis and therapy. Handbook of animal models of infection: experimental models in antimicrobial chemotherapy.

[B14] Oh S, McCaffrey JM, Eichelberger MC (2000). Dose-dependent changes in influenza virus-infected dendritic cells result in increased allogeneic T-cell proliferation at low, but not high, doses of virus. J Virol.

[B15] Epstein RA, Epstein MA, Haddad GG, Mellins RB (1980). Practical implementation of the barometric method for measurement of tidal volume. J Appl Physiol.

[B16] Mitzner W, Tankersley C (2003). Interpreting Penh in mice. J Appl Physiol.

[B17] Bhutani VK, Rubenstein SD, Shaffer TH (1981). Pressure-volume relationships of tracheae in foetal newborn and adult rabbits. Respir Physiol.

[B18] Croteau JR, Cook CD (1961). Volume-pressure and length-tension measurements in human tracheal and bronchial segments. J Appl Physiol.

[B19] McFawn PK, Mitchell HW (1997). Bronchial compliance and wall structure during development of the immature human and pig lung. Eur Respir J.

[B20] Frappell PB, MacFarlane PM (2005). Development of mechanics and pulmonary reflexes. Respir Physiol Neurobiol.

[B21] Sabogal C, Auais A, Napchan G, Mager E, Zhou BG, Suguihara C, Bancalari E, Piedimonte G (2005). Effect of respiratory syncytial virus on apnea in weanling rats. Pediatric Research.

[B22] Peng W, Zhuang J, Harrod KS, Xu F (2007). Respiratory syncytial virus infection in anesthetized weanling rather than adult rats prolongs the apneic responses to right atrial injection of capsaicin. J Appl Physiol.

[B23] Paisley JW, Bruhn FW, Lauer BA, McIntosh K (1978). Type A2 influenza viral infections in children. Am J Dis Child.

[B24] Martinez FD (1991). Sudden infant death syndrome and small airway occlusion: facts and a hypothesis. Pediatrics.

[B25] Goldman MD, Grimby A, Mead J (1976). Mechanical work of breathing derived from rib cage and abdominal V-P partitioning. J Appl Physiol.

[B26] Mortola JP, Saetta M, Fox G, Smith B, Weeks S (1985). Mechanical aspects of chest wall distortion. J Appl Physiol.

[B27] Fisher JA, Mortola JP, Smith JB, Fox GS, Weeks S (1982). Respiration in newborns: development of the control of breathing. Am Rev Respir Dis.

[B28] Mortola JP, Milic-Emili J, Noworaj A, Smith B, Fox G, Weeks S (1983). Muscle pressure and flow during expiration in infants. Am Rev Respir Dis.

[B29] Van Schaik SM, Obot N, Enhorning G, Hintz K, Gross K, Hancock GE, Stack AM, Welliver RC (2000). Role of Interferon gamma in the pathogenesis of primary respiratory syncytial virus infection in BALB/c mice. J Med Virol.

